# Chitosan Increases Tomato Root Colonization by *Pochonia chlamydosporia* and Their Combination Reduces Root-Knot Nematode Damage

**DOI:** 10.3389/fpls.2017.01415

**Published:** 2017-09-01

**Authors:** Nuria Escudero, Federico Lopez-Moya, Zahra Ghahremani, Ernesto A. Zavala-Gonzalez, Aurora Alaguero-Cordovilla, Caridad Ros-Ibañez, Alfredo Lacasa, Francisco J. Sorribas, Luis V. Lopez-Llorca

**Affiliations:** ^1^Laboratory of Plant Pathology, Department of Marine Sciences and Applied Biology, Multidisciplinary Institute for Environmental Studies – Ramón Margalef, University of Alicante Alicante, Spain; ^2^Departament d’Enginyeria Agroalimentària i Biotecnologia, Universitat Politècnica de Catalunya Castelldefels, Spain; ^3^Instituto Murciano de Investigación y Desarrollo Agrario y Alimentario Murcia, Spain

**Keywords:** endophytic colonization, nematophagous fungi, root-knot nematodes, suppressive soil, *Solanum lycopersicum*

## Abstract

The use of biological control agents could be a non-chemical alternative for management of *Meloidogyne* spp. [root-knot nematodes (RKN)], the most damaging plant-parasitic nematodes for horticultural crops worldwide. *Pochonia chlamydosporia* is a fungal parasite of RKN eggs that can colonize endophytically roots of several cultivated plant species, but in field applications the fungus shows a low persistence and efficiency in RKN management. The combined use of *P. chlamydosporia* with an enhancer could help its ability to develop in soil and colonize roots, thereby increasing its efficiency against nematodes. Previous work has shown that chitosan enhances *P. chlamydosporia* sporulation and production of extracellular enzymes, as well as nematode egg parasitism in laboratory bioassays. This work shows that chitosan at low concentrations (up to 0.1 mg ml^-1^) do not affect the viability and germination of *P. chlamydosporia* chlamydospores and improves mycelial growth respect to treatments without chitosan. Tomato plants irrigated with chitosan (same dose limit) increased root weight and length after 30 days. Chitosan irrigation increased dry shoot and fresh root weight of tomato plants inoculated with *Meloidogyne javanica*, root length when they were inoculated with *P. chlamydosporia*, and dry shoot weight of plants inoculated with both *P. chlamydosporia* and *M. javanica*. Chitosan irrigation significantly enhanced root colonization by *P. chlamydosporia*, but neither nematode infection per plant nor fungal egg parasitism was affected. Tomato plants cultivated in a mid-suppressive (29.3 ± 4.7% RKN egg infection) non-sterilized clay loam soil and irrigated with chitosan had enhanced shoot growth, reduced RKN multiplication, and disease severity. Chitosan irrigation in a highly suppressive (73.7 ± 2.6% RKN egg infection) sterilized-sandy loam soil reduced RKN multiplication in tomato. However, chitosan did not affect disease severity or plant growth irrespective of soil sterilization. Chitosan, at an adequate dose, can be a potential tool for sustainable management of RKN.

## Introduction

Plant-parasitic nematodes are a major problem for agriculture, causing crop losses of ca. $157 billion yearly (Abad et al., 2008). Among these, root-knot nematodes (RKN, *Meloidogyne* spp.) are the most damaging for vegetable crops. *Meloidogyne arenaria*, *M. incognita*, and *M. javanica* are the most widely distributed species affecting horticultural crops worldwide (Sikora and Fernández, 2005). Management of RKN has been mainly based on the use of chemical nematicides (Talavera et al., 2012), but these have been banned or restricted due to their harmful effects on the environment, as well as on wildlife and human health. Consequently, there is an urgent need for environmentally friendly and effective alternatives for RKN management, such as the use of biocontrol agents. To this respect, nematophagous fungi represent the most diverse group of nematode antagonists (Stirling, 2014).

*Pochonia chlamydosporia* Goddard (Zare and Gams; syn. *Metacordyceps chlamydosporia*) is a fungal parasite of nematodes eggs (Kerry and Hirsch, 2011). This fungus is a key component of soils suppressive to cyst (Kerry, 1982; Kerry et al., 1984) and RKN (Bent et al., 2008; Giné et al., 2016). *P. chlamydosporia* has been found to be able to colonize endophytically roots of *Arabidopsis* (Zavala-Gonzalez et al., 2016), barley (Monfort et al., 2005; Maciá-Vicente et al., 2009), potato (Manzanilla-Lopez et al., 2011), and tomato (Bordallo et al., 2002). Some isolates can also induce plant growth and reduce flowering time in tomato (Zavala-Gonzalez et al., 2015). The fungus can elicit plant defense mechanisms (Larriba et al., 2015). Jasmonate modulates root colonization (Zavala-Gonzalez et al., 2016) and influences the biocontrol capacity of *P. chlamydosporia* (Vieira Dos Santos et al., 2014). However, the presence of the fungus in roots decreases with time after inoculation (Maciá-Vicente et al., 2009; Escudero and Lopez-Llorca, 2012). Consequently, the ability of the fungus to control RKN populations could also be compromised because RKN are embedded in roots or near the root surface.

Previous studies show that a single application of *P. chlamydosporia* at a rate of 5000 chlamydospores per gram of soil can reduce damage by *M. incognita* on tomato growing in pot experiments (Bourne and Kerry, 1999; Atkins et al., 2003; Yang et al., 2012). In contrast, multiple applications of the fungus up to 1.2 10^7^ chlamydospores per plant were necessary to reduce RKN damage in the field (Sorribas et al., 2003). Attempts to increase the efficacy of RKN management in field conditions combining the application of *P. chlamydosporia* with the nematicides oxamyl (Tzortzakakis, 2000; Verdejo-Lucas et al., 2003) or fosthiazate (Tobin et al., 2008) were not successful. In this work, we test *P. chlamydosporia* combined with a 70 kDa chitosan for RKN management. Chitosan is a deacetylated and soluble form of chitin that is not toxic to plants, animals, and mammalian cells (Lopez-Moya et al., 2015). Chitosan is also biodegradable and environmentally safe (Kumar, 2000). Chitin and chitosan elicit plant defenses inhibitory to pathogenic fungi and bacteria (Chittenden and Singh, 2009; López-Mondéjar et al., 2012). Chitosan reduces RKN infection and disease severity on tomato (Khalil and Badawy, 2012). Chitosan increases *P. chlamydosporia* sporulation (Palma-Guerrero et al., 2008) and expression of VCP1, the main serine protease (Palma-Guerrero et al., 2010a) used by the fungus to parasitize nematode eggs (Escudero et al., 2016). Consequently, chitosan increases *M. javanica* egg parasitism by *P. chlamydosporia* in laboratory bioassays (Escudero et al., 2016). Thus, chitosan at an adequate dose can be a promising candidate to safely enhance effectiveness of *P. chlamydosporia* against RKN. Thus, the aim of this work was to determine the effect of chitosan on: (i) viability and germination of *P. chlamydosporia* chlamydospores; (ii) tomato plant growth; (iii) the tritrophic interaction tomato–*M. javanica*–*P. chlamydosporia* in micropots; and (iv) RKN multiplication in pot experiments using soils in which *P. chlamydosporia* occurs naturally. Our final goal was to define a concentration for chitosan irrigation compatible with tomato and *P. chlamydosporia* development for sustainable management of RKN.

## Materials and Methods

### Fungal Isolate, Root-Knot Nematode Populations, Plant Materials, and Chitosan

The strain of *P. chlamydosporia* Pc123 (ATCC MYA-4875; CECT 20929) used in this study was isolated from infected *Heterodera avenae* eggs collected in SW Spain (Olivares-Bernabeu and Lopez-Llorca, 2002). The fungus was maintained in corn meal agar (CMA; Becton Dickinson and Company, United States) at 25°C in the dark for mycelial growth. For chlamydospore production, mycelial plugs from the edge of 21-day-old fungal colonies were transferred to Vogel’s solid medium (1× Vogel’s salts, 2% sucrose, and 1.5% technical agar) and incubated at 25°C. After 4 weeks, chlamydospores were then extracted following the Kerry and Bourne (2002) procedure.

Root-knot nematode populations belonging to *M. javanica* or *M. incognita* were used in micropot and pot experiments, respectively. The *M. javanica* population was isolated from infected carnation cultivated in Chipiona (S Spain). The *M. incognita* population was isolated from tomato cultivated in Viladecans (NE Spain). Both RKN populations were maintained on susceptible tomato cultivars to produce enough inoculum for experiments. Nematode eggs were extracted by macerating infected tomato roots in 0.5% (v/v) NaClO as in Hussey and Barker (1973). Eggs were placed in Baermann trays (Whitehead and Hemming, 1965) to obtain nematode juveniles (J2).

Susceptible tomato cv. Marglobe was used in micropot experiments. Seeds were surface-sterilized with 10% (v/v) NaClO, plated on Petri dishes with germination medium (Bordallo et al., 2002), and incubated for 1 day at 4°C to favor seed stratification, followed by 5 days at 25°C in the dark and 4 additional days at 16 h:8 h (light:dark) photoperiod. Afterward, tomato plantlets were transplanted individually in 150 ml sterile cylindrical micropot containing 70 cm^3^ of sterilized sand. Susceptible tomato cv. Durinta with three true developed leaves provided by Planters Rovira (Barcelona, Spain) was used in pot experiments.

Chitosan with a deacetylation degree of 80.5% and 70 kDa molecular weight was obtained from Marine BioProducts GmbH (Bremerhaven, Germany) and prepared as in Palma-Guerrero et al. (2008). Chitosan was dialyzed for salt removal against distilled water for laboratory and micropot experiments.

### Effect of Chitosan on *P. chlamydosporia* Chlamydospores Viability and Mycelia Growth

Suspensions of 10^5^ chlamydospore ml^-1^ including 1, 2, 3, or 4 mg ml^-1^ chitosan (final concentration) were used to assess the effect of chitosan on chlamydospore viability. The suspensions were incubated at room temperature for 4 h and then stained with 5 mg ml^-1^ propidium iodide (PI) which penetrates plasma membrane from dead cells labeling them red (nucleic acid staining), while living cells remain unstained. Fluorescence was recorded with a Leica TCS-SP2 laser-scanning confocal microscope, using 488 and 560 nm excitation and detection wavelengths, respectively (Oparka and Read, 1994; Hickey et al., 2005). Chlamydospore suspensions mixed with sterilized distilled water or hydrogen peroxide were used as negative and positive controls, respectively. Thirty chlamydospores were assessed per treatment.

Chlamydospore germination assays were carried out on 10-well microscope slides (Waldemar Knittel). Each well was filled with 2.5 × 10^4^ chlamydospores and aliquots of chitosan solutions to reach a final concentration of either 0, 0.01, 0.05, 0.1, 0.5, 1, and 2 mg ml^-1^ in a final volume of 25 μl. Higher chitosan concentrations were discarded because chlamydospore viability was compromised. Slides were incubated in moist chambers at room temperature in the dark for 24 h. Percentage of germination in random samples of 200 chlamydospores per well was then scored in an Olympus BH-2 microscope. A chlamydospore was considered germinated when the germ tube length was 1.5 times the chlamydospore diameter (Plascencia-Jatomea et al., 2003). Three slides per treatment were scored and the experiment was carried out twice.

The effect of chitosan on mycelium developing from chlamydospores was carried out as described by Lopez-Moya et al. (2015). Two-hundred microliter aliquots of either 0, 0.01, 0.05, 0.075, 0.1, 1, or 2 mg ml^-1^ chitosan solutions (final concentration) mixed with 2.5 10^3^ chlamydospores were dispensed in 96-well microtiter plates (Sterilin Ltd., Newport, United Kingdom). Growth was estimated daily for 8 days by measuring the optical density at 490 nm (OD_490_) in a GENios (Tecan, Männedorf, Switzerland) spectrofluorometer. Each treatment was evaluated in four wells and the experiment was carried out three times.

### Effect of *P. chlamydosporia* and Chitosan on Tomato Growth

Tomato cv. Marglobe plantlets in micropots containing sterilized sand were singly inoculated with four 5-mm-diameter plugs taken from the edge of a 20-day-old *P. chlamydosporia* colony grown on CMA. Corn meal agar plugs without fungus were used in non-inoculated plants. The fungal inoculum was placed 1 cm deep and mixed with the substrate as in Macia-Vicente et al. (2008). Plantlets were irrigated daily with a 0.1× Gamborg’s B5 nutrient solution amended with final chitosan concentrations of either 0, 0.01, 0.05, 0.075, 0.1, and 0.3 mg ml^-1^. Plantlets were randomly distributed in the growth chamber (Fitoclima 10000EHVP) and maintained at 25°C, 65% relative humidity, and 16 h:8 h (light:dark) photoperiod. Dry shoot weight (DSW), maximum shoot length (MSL), fresh root weight (FRW), and maximum root length (MRL) were scored per plant at 10, 20, and 30 days after fungal inoculation. Each fungus-chitosan concentration and sampling time combination was replicated 10 times, and the experiment was performed twice.

### Effect of Chitosan on the Tritrophic Interaction Tomato–*M. javanica*–*P. chlamydosporia*

Tomato seeds were germinated, inoculated with *P. chlamydosporia* (Pc) and irrigated with 0.1 mg ml^-1^ chitosan (0.1chi) as described. Twenty-five-day-old plants were inoculated with two *M. javanica* J2 (RKN) per cm^3^ of substrate. Treatments containing Pc were re-inoculated with 5000 chlamydospores g^-1^ substrate 30 dai (Kerry and Bourne, 2002). Micropots were placed randomly in a growth chamber (Fitoclima 10000EHVP) at 25°C, 65% relative humidity, and 16 h:8 h (light:dark) photoperiod. After 56 days, DSW, MSL, FRW, and MRL per plant were assessed. Egg masses of *M. javanica* per plant were counted after staining with 1% eosin yellowish hydroalcoholic solution (Panreac) (Roberts et al., 1990). The experiment consisted of four treatments: Tomato (To)+RKN, To+RKN+0.1chi, To+RKN+Pc, and To+RKN+ Pc +0.1chi, with 10 replicates each. The experiment was performed twice (80 plants in total).

Fungal egg parasitism was assessed as in Giné et al. (2013). Briefly, at the end of the experiment, 30 egg masses per treatment were handpicked from tomato roots and divided into six subsamples in 1000 μl sterile distilled water each. Eggs were dispersed from egg masses using a pestle, and 333 μl aliquots of the egg suspensions were spread onto Petri dishes containing a growth-restricting medium for *P. chlamydosporia* slightly modified from Lopez-Llorca and Duncan (1986) (50 μg ml^-1^ streptomycin, 50 μg ml^-1^ chloramphenicol, 50 μg ml^-1^ chlortetracycline, 50 μg ml^-1^ rose bengal, 0.5 % triton X-100, and 1.5% agar). Petri dishes were incubated at 25°C in the dark. The number of parasitized eggs was recorded 96 h after plating using a dissecting microscope. Eggs were considered parasitized when hyphae grew from inside. The percentage of parasitism was calculated as the proportion of the number of parasitized eggs respect to the total number of eggs plated.

Tomato root colonization by *P. chlamydosporia* was estimated using real time quantitative PCR (qPCR) as in Escudero and Lopez-Llorca (2012). Briefly, DNA was extracted independently from three tomato roots per treatment. Primers for *P. chlamydosporia* detection were VCP1q_F (5′–3′GCCATCGTTGAGCAGCAG) and VCP1q_R (5′–3′ACCGTGACCGTCGTTGTTCT). qPCR reactions were performed using the FastStart Universal SYBR Green Master (Roche) mix in a final volume of 10 μl, containing 100 ng of total DNA and 0.25 μM of each primer. Reactions were performed in triplicate in a Thermal Cycling StepOne Plus (Applied Biosystems) using the following thermal cycles: 95°C for 10 min followed by 40 cycles of 95°C for 15 s and 60°C for 45 s. Pc123 genomic DNA dilutions were used to define a calibration curve from 30 ng to 3 pg. After each run, a dissociation curve was acquired to check amplification specificity. Fungal DNA was referred to total DNA.

### Effect of Chitosan on RKN Multiplication on Tomato in Soils in Which *P. chlamydosporia* Occurs Naturally

Chitosan at 0.1 mg ml^-1^ was applied weekly in a pot experiment using two agricultural soils from NE Spain, henceforth referred to as M10.41 and M10.56. *P. chlamydosporia* and other fungal egg parasites of RKN occur naturally in both soils (Giné et al., 2013). Soil from site M10.41 is clay loam (33% sand, 29% clay, and 38 silt); pH 8.2; electric conductivity 516 μS/cm; 4.4 organic matter (w/w) content, and 33% RKN eggs produced in zucchini–squash in July 2015 were parasitized by *P. chlamydosporia*. Soil from site M10.56 is sandy loam (53% sand, 18% clay, and 29 silt); pH 8.3; electric conductivity 415 μS/cm; 4.3 organic matter (w/w) content, and 70% RKN eggs produced on tomato in July 2015 were parasitized by *P. chlamydosporia*. Soil samples were taken in February 2016 and processed as in Giné et al. (2016). Each soil was sieved through a 4-mm mesh and split into two subsamples. A subsample from each soil was autoclave-sterilized for 1 h at 121°C. The sterilization process was repeated after 24 h. The remaining soil subsamples were stored at 4°C. Sterilized and non-sterilized soils were mixed with sterile sand (1:1; v:v) before being used as plant substrate in pots, to favor aeration and root development. In both soils, *Meloidogyne* sp. population densities were determined from two 500 cm^3^ subsamples using Baermann trays after a week of incubation at 25°C. Tomato cv. Durinta plantlets with three true developed leaves were individually transplanted to 3 l pots and inoculated with *M. incognita* J2. *Meloidogyne* spp. J2 extracted from each non-sterilized soil were taken into account to inoculate a final amount of 3000 J2 per pot. The experiment included four treatments per agricultural soil: (i) sterile soil mixture, (ii) sterile soil mixture + 0.1 mg ml^-1^ chitosan irrigation, (iii) non-sterile soil mixture with no-chitosan irrigation, and (iv) non-sterile soil mixture + 0.1 mg ml^-1^ chitosan irrigation. Each treatment was replicated 10 times.

Plants were maintained in greenhouse conditions and irrigated as required. Those from the chitosan treatment were irrigated weekly with approximately 150 ml of 0.1 mg ml^-1^ chitosan per plant. The experiment was carried out in 2016, from April 19 to July 4. At the end of the experiment, plants were removed from pots and DSW and RFW were determined as described. Galling index was estimated by the 0–10 Zeck (1971) scale, where 0 is a non-galled root and 10 is a completely galled root system. RKN eggs were extracted from roots as in Hussey and Barker (1973). J2 were extracted from soil using Baermann trays and their number per plant scored. Percentage of fungal egg parasitism was assessed 24 and 48 h after plating.

### Statistical Analyses

Statistical analyses were performed in R (v. 3.1.2) (R Core Team, 2014). Variables were log_10_(*x* + 1) or square root(*x* + 0.5) transformed when required. Homoscedasticity was checked using Levene’s test and normality using Shapiro–Wilk’s test. Differences between treatments were tested by Dunnett’s or Student’s *t*-tests (*p* < 0.05). All data are reported as mean ± standard error (SE).

## Results

### Effect of Chitosan on *P. chlamydosporia* Chlamydospores Viability and Mycelia Growth

**FIGURE 1:**
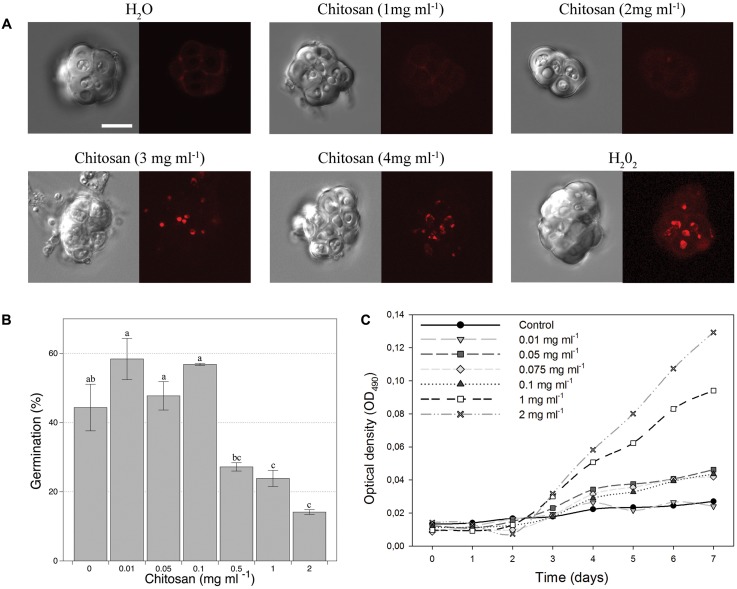
Effect of chitosan on *Pochonia chlamydosporia* chlamydospores. **(A)** Viability. DIC images on the right and fluorescence images of the same chlamydospores on the left. Dead chlamydospores red stained with 5 μg ml^-1^ propidium iodide indicate full plasma membrane permeabilization (cell death). Controls: H_2_O (negative), H_2_O_2_ (positive). Bar = 10 μm. **(B)** Germination. **(C)** Growth kinetics from chlamydospores as initial inoculum in chitosan as sole nutrient. Chitosan concentrations are shown in each graph and asterisks indicate significant differences (*p-*value < 0.05) vs. control (no chitosan).

Chitosan solutions at concentrations up to 2 mg ml^-1^ did not affect viability of chlamydospores (**Figure 1A**). Higher chitosan concentrations caused chlamydospore death (PI staining), just as H_2_O_2_ treatments. Chitosan at concentrations up to 0.1 mg ml^-1^ did not affect chlamydospore germination (**Figure 1B**). Higher concentrations significantly reduced germination. Two milligrams per milliliter chitosan reduced ca. 70% germination respect to untreated controls, but favored mycelial growth after 3 days of incubation. Maximum mycelia growth (measured as OD_490_) was obtained with 2 mg ml^-1^ chitosan. These results suggest that *P. chlamydosporia* uses chitosan as a nutrient source. Chitosan concentrations between 0.05 and 0.1 mg ml^-1^ did not affect viability/germination of chlamydospores and improved mycelial growth compared to the control (**Figure 1C**).

### Effect of *P. chlamydosporia* and Chitosan on Tomato Growth

The effect of *P. chlamydosporia* inoculation on tomato plants, not irrigated with chitosan was first evaluated. *P. chlamydosporia* increased both DSW and FRW 10 days after inoculation (Supplementary Table 1), but this effect was not sustained over time. However, fungal inoculation increased shoot length of tomato plants at 30 days (**Table 1**).

**Table 1:** Effect of the concentration of chitosan alone applied weekly by irrigation or combined with *Pochonia chlamydosporia* (Pc) on dry shoot weight (DSW), maximum shoot length (MSL), fresh root weight (FRW), and maximum root length (MRL) of tomato plants after 30 days.

Chitosan concentration	DSW (g)		MSL (cm)		FRW (g)		MRL (cm)	
				
[mg ml^-1^]	- Pc	+ Pc	- Pc	+ Pc	- Pc	+ Pc	- Pc	+ Pc
0	0.16 ± 0.01	0.19 ± 0.01	9.17 ± 0.44	10.45 ± 0.32^∗^	0.92 ± 0.07	1.06 ± 0.09	10.47 ± 0.35	12.31 ± 0.87
0.01	0.16 ± 0.01	0.19 ± 0.01	7.89 ± 0.50	**7.88 ± 0.38**	1.12 ± 0.12	1.30 ± 0.04	12.57 ± 0.68	11.72 ± 0.82
0.05	**0.21 ± 0.01**	0.20 ± 0.01	8.04 ± 0.10	**8.17 ± 0.22**	**2.34 ± 0.09**	**1.37 ± 0.08**	12.13 ± 0.46	12.45 ± 0.42
0.075	0.18 ± 0.01	0.20 ± 0.01	8.25 ± 0.39	**8.44 ± 0.23**	1.07 ± 0.05	1.28 ± 0.08	**14.11 ± 0.59**	11.95 ± 0.43
0.1	0.17 ± 0.01	0.17 ± 0.01	8.02 ± 0.25	**7.75 ± 0.15**	**1.27 ± 0.09**	**1.42 ± 0.06**	**13.6 ± 0.96**	12.87 ± 0.82
0.3	**0.13 ± 0.01**	**0.08 ± 0.01**	7.41 ± 0.30	**6.85 ± 0.44**	0.84 ± 0.04	**0.50 ± 0.05**	10.56 ± 0.61	**8.24 ± 0.47**


*Values are means ± SE (*n* = 10). Data in bold in the same column differ from 0 mg ml^-*1*^ (Dunnett’s test; *p*-value < 0.05), asterisk indicates differences due to *P. chlamydosporia* inoculation for no-chitosan irrigation treatments (Student’s *t*-test; *p*-value < 0.05).*

Chitosan irrigation (0.01–0.075 mg ml^-1^) of tomato plants uninoculated with *P. chlamydosporia* promoted shoot growth (DSW) at 10 days, but the effect was lost with time (**Table 1** and Supplementary Tables 1, 2). The same doses promoted root growth (FRW) at 20 days and to a less extent (0.05 and 0.1 mg ml^-1^ only) at 30 days (Supplementary Table 2 and **Table 1**). However, chitosan applied at 0.3 mg ml^-1^ reduced tomato growth (**Table 1**).

*Pochonia chlamydosporia* combined with low chitosan concentrations (0.01–0.1 mg ml^-1^) promoted root growth (FRW) after 20 days, and to a less extent (0.05 and 0.1 mg ml^-1^) after 30 days. On the contrary, *P. chlamydosporia* combined with the largest chitosan concentration (0.3 mg ml^-1^) reduced shoot and root weight, MRL, and MSL 30 days after fungal inoculation and chitosan application (**Table 1**). Shoot length was very sensitive to *P. chlamydosporia* combined with chitosan, because all treatments abolished the MSL promotion obtained with the fungus inoculated alone (no chitosan) after 30 days (**Table 1**). The results obtained from this section selected 0.1 mg ml^-1^ of chitosan irrigation for further experiments.

### Effect of Chitosan on the Tritrophic Interaction Tomato–*M. javanica*–*P. chlamydosporia*

**FIGURE 2:**
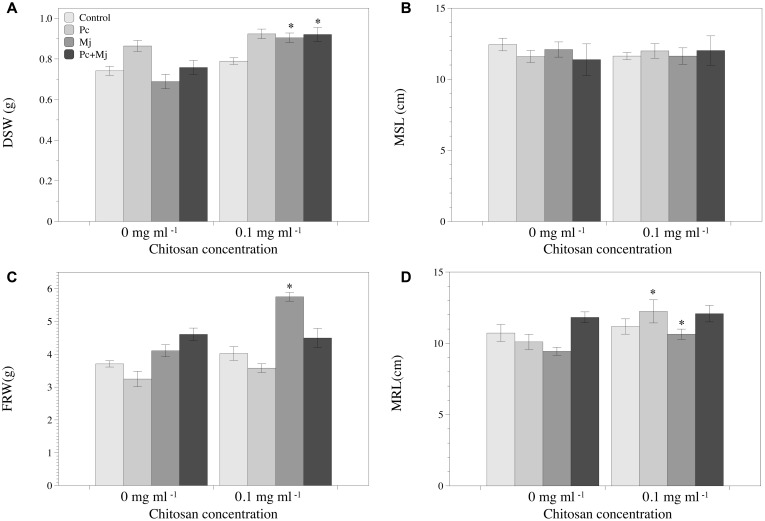
Effect of *P. chlamydosporia*, *Meloidogyne javanica*, and chitosan on growth of tomato plantlets. **(A)** Dry shoot weight (DSW), **(B)** maximum shoot length (MSL), **(C)** fresh root weight (FRW), and **(D)** maximum root length (MRL). Asterisks indicate differences in the chitosan treatments respect to those without chitosan (*p-*value < 0.05). C, control (non-inoculated); Pc, plants inoculated with *P. chlamydosporia* only; Mj, plants inoculated with *M. javanica* only; and Pc+Mj, plants inoculated with both *P. chlamydosporia* and *M. javanica*.

**FIGURE 3:**
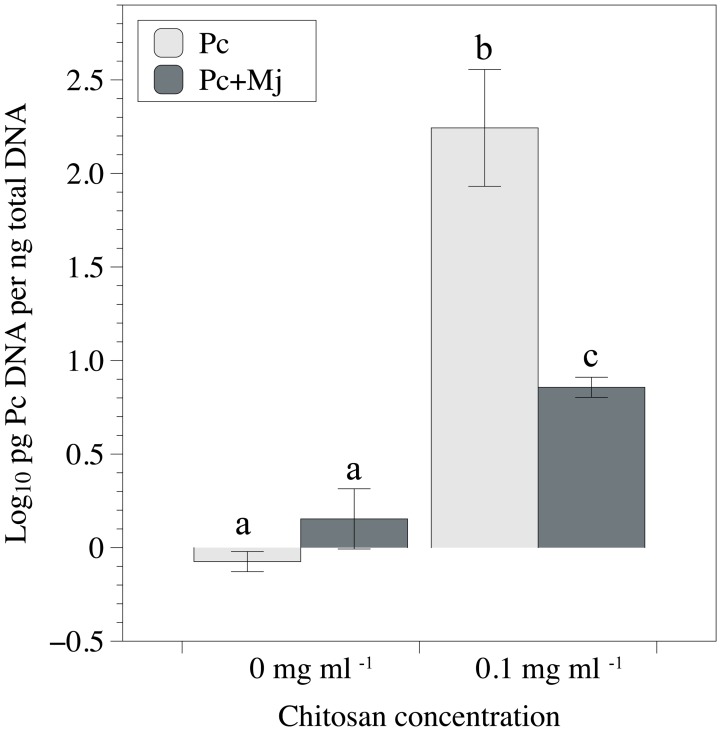
Effect of chitosan irrigation and *M. javanica* inoculation on root colonization of tomato plants by *P. chlamydosporia* estimated by qPCR. Pc, plants inoculated with *P. chlamydosporia* only and Pc+Mj, plants inoculated with both *P. chlamydosporia* and *M. javanica*. Different letters indicate statistical differences (*p*-value < 0.05).

Chitosan irrigation increased DSW (**Figure 2A**) and FRW and root length (**Figures 2C,D**) in tomato plants inoculated with *M. javanica*. Chitosan also promotes root length in plants inoculated with *P. chlamydosporia* (**Figure 2D**), and DSW in plants inoculated with both *P. chlamydosporia* and *M. javanica* (**Figure 2A**). Differences in maximum shoot length were not found (**Figure 2C**). The number of egg masses per plant was not influenced by chitosan irrigation irrespective of fungal inoculation (data not shown). Fungal egg parasitism was low (2.3–7.2%) and did not differ with chitosan irrigation. Chitosan irrigation significantly (*p* < 0.05) enhanced tomato root colonization by *P. chlamydosporia* (**Figure 3**) by 20-fold. In RKN treatments, chitosan irrigation also significantly increases the colonization of roots by the fungus but to a lesser extent than roots with no RKN.

### Effect of Chitosan on RKN Multiplication on Tomato in Soils in Which *P. chlamydosporia* Occurs Naturally

**FIGURE 4:**
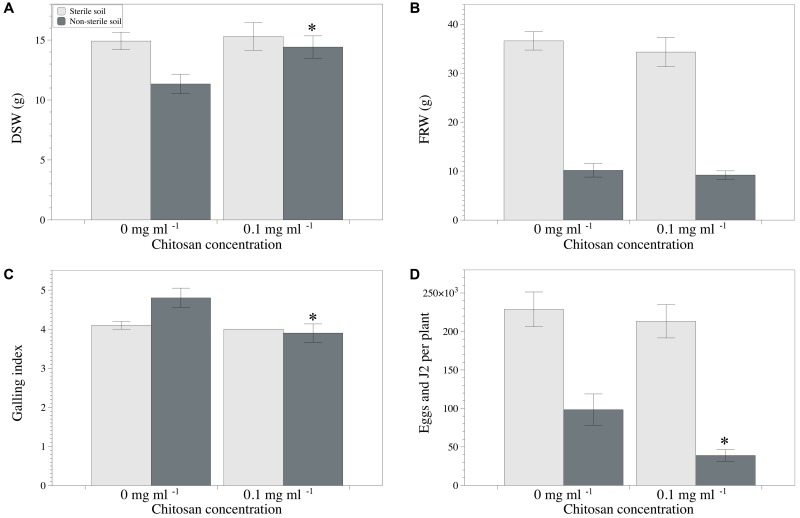
Effect of chitosan irrigation on RKN (*Meloidogyne* spp.) in tomato plants grown in M10.41 clay soil with natural moderate suppression to RKN. **(A)** DSW, **(B)** FRW, **(C)** Galling index, and **(D)** total nematodes per plant. Asterisks show statistical differences between control and chitosan treatments (*p*-value < 0.05).

Chitosan irrigation of tomato plants cultivated in the non-sterilized clay loam soil M10.41 enhanced (*p* < 0.05) shoot growth, reduced galling index, and RKN multiplication (**Figures 4A,C,D**), but no effect was observed in the sterilized soil. No differences were found for fresh root weight (**Figure 4B**). Fungal egg parasitism was 36.6 ± 3.3% and 29.3 ± 4.7% in chitosan irrigated and non-irrigated non-sterilized soil, respectively. *P. chlamydosporia* was the only fungus identified parasitizing nematode eggs.

**FIGURE 5:**
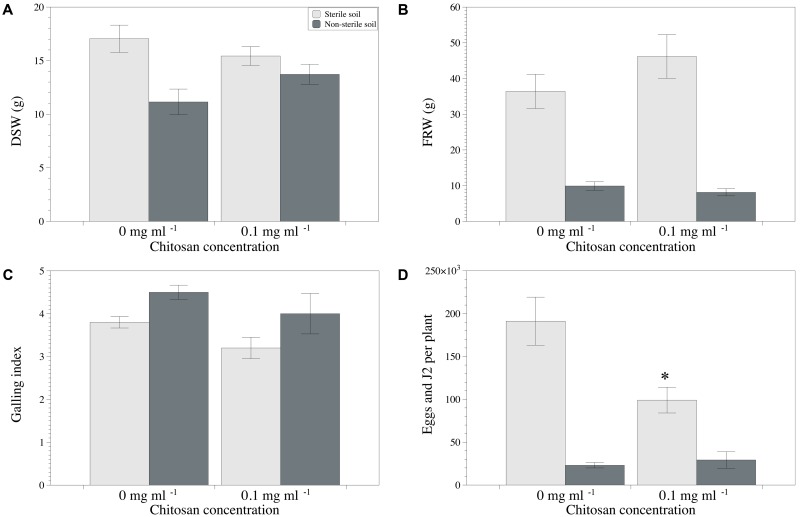
Effect of chitosan irrigation on RKN (*Meloidogyne* spp.) in tomato plants grown in M10.56 sandy soil with natural high suppression to RKN. **(A)** DSW, **(B)** FRW, **(C)** Galling index, and **(D)** total nematodes per plant. Asterisks show statistical differences between control and chitosan treatments (*p*-value < 0.05).

Regarding the sandy loam soil M10.56, chitosan irrigation reduced RKN multiplication (sterilized soil only), but had not effect (*p* < 0.05) on disease severity or plant growth irrespective of soil sterilization (**Figures 5A–C**). No differences were found for fresh root weight (**Figure 5B**). Fungal egg parasitism was 45.7 ± 6.0% and 73.7 ± 2.6% in the non-sterile soil irrigated and non-irrigated with chitosan, respectively. *P. chlamydosporia* was again the only fungus parasitizing the nematode eggs. No fungal egg parasitism was detected on eggs produced on tomato plants grown in the sterile soil mixtures from both sites.

## Discussion

Chitosan enhances *P. chlamydosporia* sporulation, protease induction (Palma-Guerrero et al., 2010a,b), appressorium differentiation, and nematode egg parasitism in laboratory bioassays (Escudero et al., 2016). However, little is known about the effect of chitosan on the tritrophic interaction Plant–RKN–*P. chlamydosporia*, or on RKN suppressiveness when applied to agricultural soils containing natural nematode antagonists.

The first aim of this work was to determine the effect of a 70 kDa chitosan on the viability of *P. chlamydosporia* chlamydospores. These resistant spores are used as inoculum for nematode management and consistently found in suppressive soils (Kerry and Bourne, 2002). Viability of *P. chlamydosporia* chlamydospores was kept at concentrations of chitosan up to 2 mg ml^-1^ which are toxic for several fungal species causing soil-borne diseases, such as *Fusarium oxysporum* f. sp. *radicis-lycopersici, Verticillium dahliae*, *Rhizoctonia solani* (Allan and Hadwiger, 1979; Palma-Guerrero et al., 2008; Xing et al., 2015). Thus, soil irrigation with chitosan could inhibit soilborne fungal pathogens without affecting *P. chlamydosporia* chlamydospores.

Our results show that mycelium of *P. chlamydosporia* derived from chlamydospores can grow with chitosan as the only carbon source. The large expansion of GH75 chitosanase family in the genome of *P. chlamydosporia* (Aranda-Martinez et al., 2016) could explain this feature.

This is, to the best of our knowledge, the first time *P. chlamydosporia* has been applied combined with chitosan against RKN in micropot experiments. Chitosan at 0.1 mg ml^-1^ increased the growth of *M. javanica* infested plants respect to those inoculated the nematode only, as previously reported by Vasyukova et al. (2001). Chitosan combined with *P. chlamydosporia* promoted root colonization by the fungus respect to plants inoculated with *P. chlamydosporia* alone. Chitosan, perhaps acting as an alternative carbon source, is involved in the increase of tomato root colonization by *P. chlamydosporia* as it found in this work. This could help improving rhizosphere competence of the fungus which decreases with time (Maciá-Vicente et al., 2009; Escudero and Lopez-Llorca, 2012). The mechanism involved is unknown but should be further investigated. To this respect, chitosan is a poor substrate for chitinases and a weak inducer of plant immune response (Sanchez-Vallet et al., 2015).

*Pochonia chlamydosporia*, chitosan, or its combination did not affect nematode infection and development in roots. Therefore RKN juveniles, under our experimental conditions, could infect and develop into mature females laying eggs. In addition, neither *P. chlamydosporia*, chitosan, nor its combination affected fungal egg parasitism. The ability of chitosan to increase root colonization by *P. chlamydosporia* found in this study should be used in future work to increase plant tolerance/resistance to RKN.

Chitosan irrigation of agricultural soils containing natural nematode antagonists had different effects depending on soil properties such as the resident microbiota and soil texture. In the non-sterilized clay loam M10.41 soil, chitosan promoted plant growth and reduced the number of nematodes per plant. These effects were associated with the nematode antagonistic microbiota but mechanisms other than egg-parasitism (only slightly enhanced) were probably involved. The differential effect of chitosan on soil microorganisms could induce rearrangements of microbial communities, which could affect the level of nematode suppression in soils (Tian et al., 2015). Accordingly, chitosan had no effect in M10.41 sterilized soil.

Chitosan irrigation reduced nematodes per plant in sterilized M10.56 sandy loam soil. However, no such effect was found in non-sterilized soil, where high parasitism of RKN egg by *P. chlamydosporia* only was recorder. These results are in concordance with those reported by Khalil and Badawy (2012) and Radwan et al. (2012) who found a reduction of RKN densities in tomato irrigated with chitosan in soils with at least 50% sand.

The present study demonstrates that chitosan applied at low rates does not affect chlamydospores and enhances mycelial growth of *P. chlamydosporia*. However, soil texture and nematode antagonistic microbiota in agricultural soils affect the performance of chitosan against RKN. Microbiota in agricultural soils putative antagonistic to RKN is diverse (Giné et al., 2016). Chitosan properties (e.g., deacetylation degrees and molecular weight) have different effects on microorganisms (Younes et al., 2014). Therefore, the interaction of these features should be analyzed for improving chitosan as a bioactivator of RKN antagonists, such as *P. chlamydosporia*.

## Author Contributions

This work was a part of the Ph.D. thesis of NE supervised by LL-L. NE design and performance of the research, data collection and data analysis, and writing of the manuscript. FL-M performance of the research, data collection, and writing of the manuscript. ZG, EZ-G, AA-C, CR-I, and AL performance of the research and technical support. FS design and performance of the research, data collection, data interpretation, and writing of the manuscript. LL-L design of the research, data interpretation, and writing of the manuscript.

## Conflict of Interest Statement

The authors declare that the research was conducted in the absence of any commercial or financial relationships that could be construed as a potential conflict of interest.
