# Supplementary material for: Chitosan Increases Tomato Root Colonization by Pochonia chlamydosporia and Their Combination Reduces Root-Knot Nematode Damage
Source: Front Plant Sci. 2017 Sep 1;8:1415. doi: 10.3389/fpls.2017.01415 (PMC5585746; doi:10.3389/fpls.2017.01415)
Supplement: Supplementary file 1 [file Table_1.DOCX]

Supplementary table 1.- Effect of the concentration of chitosan alone applied weekly by irrigation or combined with *P. chlamydosporia* (Pc) on Dry shoot weight (DSW), Maximum shoot length (MSL), Fresh root weight (FRW); Maximum root length (MRL) of tomato plants after 10 days.

| Quitosan concentration  [mg ml^-1^] | DSW (g) | | MSL (cm) | | FRW (g) | | MRL (cm) | |
| --- | --- | --- | --- | --- | --- | --- | --- | --- |
|  | - Pc | + Pc | - Pc | + Pc | - Pc | + Pc | - Pc | + Pc |
| 0 | 0.006 ± 0.001 | 0.012 ± 0.001* | 4.6 ± 0.39 | 4.93 ± 0.33 | 0.027 ± 0.003 | 0.061 ± 0.006^*^ | 8.51 ± 0.98 | 9.20 ± 0.64 |
| 0.01 | **0.010 ± 0.001** | 0.010 ± 0.001 | 4.13 ± 0.26 | 3.75 ± 0.19 | 0.055 ± 0.005 | 0.053 ± 0.005 | 12.55 ± 0.73 | 10.6 ± 1.15 |
| 0.05 | **0.013 ± 0.001** | 0.011 ± 0.001 | 4.1 ± 0.18 | 4.69 ± 0.39 | 0.066 ± 0.007 | 0.049 ± 0.007 | 9.72 ±0.73 | 9.63 ± 1.36 |
| 0.075 | **0.009 ± 0.001** | 0.009 ± 0.001 | 2.96 ± 0.24 | 4.57 ± 0.19 | 0.056 ± 0.005 | 0.041 ± 0.005 | 9.86 ±0.61 | 7.74 ±0.50 |
| 0.1 | 0.008 ± 0.001 | **0.009 ± 0.001** | 3.65 ± 0.29 | 4.34 ± 0.30 | 0.026 ± 0.003 | 0.028 ± 0.002 | 7.90 ±0.87 | 8.89 ± 0.71 |
| 0.3 | 0.0010 ± 0.001 | **0.009 ± 0.001** | 4.89 ± 0.34 | 4.28 ± 0.28 | 0.033 ± 0.003 | 0.034 ± 0.003 | 8.51 ±0.81 | 8.56 ± 1.04 |

Values are means±SE (n=10). Data in bold in the same column differ from 0 mg ml^-1^ (Dunnett’s test; *p-value*<0.05) asterisks indicate differences due to *P. chlamydosporia* inoculation for no-chitosan irrigation treatments (t-student test; *p-value*<0.05) .
