# Supplementary material for: Chitosan Increases Tomato Root Colonization by Pochonia chlamydosporia and Their Combination Reduces Root-Knot Nematode Damage
Source: Front Plant Sci. 2017 Sep 1;8:1415. doi: 10.3389/fpls.2017.01415 (PMC5585746; doi:10.3389/fpls.2017.01415)
Supplement: Supplementary file 2 [file Table_2.DOCX]

Supplementary table 2.- Effect of the concentration of chitosan alone applied weekly by irrigation or combined with *P. chlamydosporia* (Pc) on Dry shoot weight (DSW), Maximum shoot length (MSL), Fresh root weight (FRW); Maximum root length (MRL) of tomato plants after 20 days.

| Quitosan concentration  [mg ml^-1^] | DSW (g) | | MSL (cm) | | FRW (g) | | MRL (cm) | |
| --- | --- | --- | --- | --- | --- | --- | --- | --- |
|  | - Pc | + Pc | - Pc | + Pc | - Pc | + Pc | - Pc | + Pc |
| 0 | 0.066 ± 0.006 | 0.065 ± 0.009 | 7.72 ± 0.34 | 7.44 ± 0.27 | 0.26 ± 0.03 | 0.34 ± 0.04 | 9.82 ± 0.66 | 8.55 ± 0.22 |
| 0.01 | 0.081 ± 0.009 | 0.082 ± 0.006 | 7.22 ± 0.35 | 7.22 ± 0.29 | **0.55 ± 0.10** | **0.60 ± 0.04** | 9.92 ± 0.51 | 8.97 ± 0.26 |
| 0.05 | 0.083 ± 0.005 | 0.080 ± 0.007 | 7.21 ± 0.23 | 7.21 ± 0.29 | **0.68 ± 0.06** | **0.61 ± 0.08** | 12.25 ± 1.00 | 10.77 ± 0.41 |
| 0.075 | 0.064 ± 0.004 | 0.084 ± 0.008 | 7.65 ± 0.21 | 7.65 ± 0.68 | **0.48 ± 0.04** | 0.48 ± 0.04 | 11.73 ± 0.61 | 11.23 ± 0.86 |
| 0.1 | 0.045 ± 0.003 | 0.075 ± 0.010 | 7.14 ± 0.33 | 7.14 ± 0.21 | 0.27 ± 0.03 | **0.55 ± 0.06** | 10.31 ± 0.60 | 10.07 ± 0.76 |
| 0.3 | 0.060 ± 0.007 | 0.047 ± 0.004 | 6.61 ±0.35 | 6.51 ± 0.37 | 0.21 ± 0.03 | 0.21 ± 0.03 | 8.04 ± 0.75 | 10.04 ± 0.84 |

Values are means±SE (n=10). Data in bold in the same column differ from 0 mg ml^-1^ (Dunnett’s test; *p-value*<0.05).
